# Supplementary material for: Identification of photoperiod-induced specific miRNAs in the adrenal glands of Sunite sheep (Ovis aries)
Source: Front Vet Sci. 2022 Jul 22;9:888207. doi: 10.3389/fvets.2022.888207 (PMC9354845; doi:10.3389/fvets.2022.888207)
Supplement: Supplementary file 1 [file Data_Sheet_1.ZIP › Supplementary file/miRDeep2 file.zip!/miRDeep2 file/Know_Structure/SPLP42A2/oar-let-7a.pdf]

miRBase precursor : oar-let-7a
Total read count : 383254
oar-let-7a read count : 381616
remaining reads : 1638

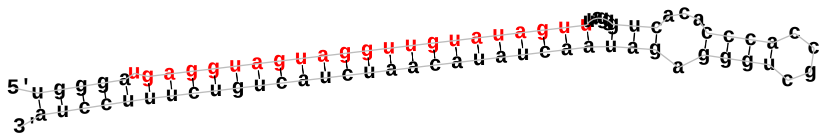

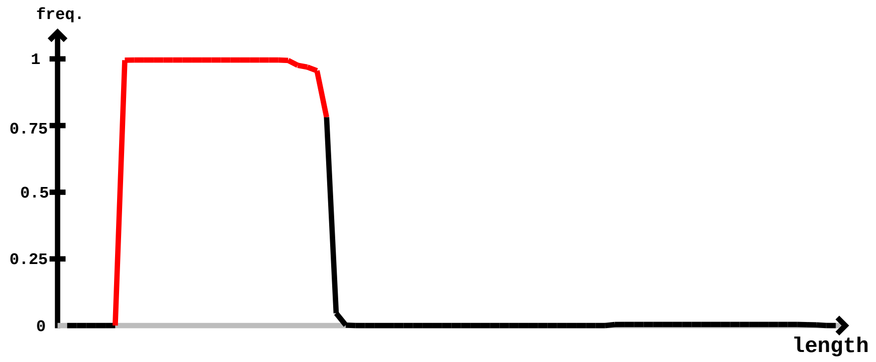

oar-let-7a

5'- ugggaugagguaguagguuguauaguuuuagggucacacccaccgcugggagauaacuauacaaucuacugucuuuccua -3' exp

(((((.(((((((((((((((((((((.....(((...((((....)))).)))))))))))))))))))))))))))))	reads	mm	sample
...gUugagguaguagguuguauagu..	1	1	seq
....augagguaguagguuguauag...	2	0	seq
....Uugagguaguagguuguauagu..	2	1	seq
....augagguaguagguuguauagu..	7	0	seq
....Uugagguaguagguuguauaguu...	8	1	seq
....augagguaguagguuguauaguu...	14	0	seq
....Uugagguaguagguuguauaguuu..	2	1	seq
.....ugagguaguaAguugua..	11	1	seq
.....ugagguUguagguugua..	1	1	seq
.....ugaAguaguagguugua..	4	1	seq
.....ugagguaguagguugua..	500	0	seq
.....ugaggCaguagguugua..	1	1	seq
.....ugagguaguagguuUua..	4	1	seq
.....ugagguagGagguugua..	84	1	seq
.....ugaAguaguagguuguau...	42	1	seq
.....ugagguaguagguuguau...	6310	0	seq
.....ugagguUguagguuguau...	5	1	seq
.....ugagguagGagguuguau...	619	1	seq
.....ugagguaguaAguuguau...	44	1	seq
.....ugagguaguagguuUuau...	12	1	seq
.....ugagguagGagguuguaua..	312	1	seq
.....ugagguaguagguuguaua..	2223	0	seq
.....ugagguaguagguuguauag...	4393	0	seq
.....ugagguagGagguuguauag...	805	1	seq
.....ugagguagGagguuguauagu..	9107	1	seq
.....ugagguaguagguuguauagu..	57700	0	seq
.....ugagguagGagguuguauaguu...	26413	1	seq
.....ugagguaguagguuguauaguu...	255078	0	seq
.....ugagguaguagguuguauaguuu..	17090	0	seq
.....ugagguaguagguuguauaguuuu...	469	0	seq
.....ugagguaguagguuguauaguuuA...	116	1	seq
.....ugagguaguagguuguauaguuuua..	9	0	seq
.....ugagguaguagguuguauaguuuuagggu..	3	0	seq
......gagguaguagguuguau...	5	0	seq
......gagguagGagguuguau...	1	1	seq

oar-let-7a

```
ugggaugagguaguagguuguauaguuuuagggucacacccaccgcugggagauaacuauacaaucuacugucuuuccua
......gagguaguaAguuguau.........................................................    1     1    seq
......gagguaguagguuguauag.......................................................    1     0    seq
......gagguagGagguuguauagu......................................................    6     1    seq
......gagguaguagguuguauagu......................................................    25    0    seq
......gagguaguagguuguauaguu.....................................................    152   0    seq
......gagguagGagguuguauaguu.....................................................    21    1    seq
......gagguaguagguuguauaguuu....................................................    5     0    seq
.......agguaguagguuguauagu......................................................    2     0    seq
.......agguaguagguuguauaguu.....................................................    4     0    seq
.......agguaguagguuguauaguuuu...................................................    2     0    seq
........gguaguagguuguauagu......................................................    2     0    seq
........gguaguagguuguauaguuu....................................................    1     0    seq
..........uaguagguuguauaguuuuaggg...............................................    1     0    seq
...........................uuagggucacacccaccgcugg...............................    3     0    seq
...........................uuagggucacacccaccgcuggg..............................    1     0    seq
...........................uuagggucacacccaccgcuggga.............................    7     0    seq
.....................................................uaacuauacaaucuacugucu......    1     0    seq
.....................................................uaacuauacaaucuacugucuuuc...    2     0    seq
.......................................................acuauacaaucuacugucuuuc...    1     0    seq
........................................................cuauacaaucuacugucu......    3     0    seq
........................................................cuauacaaucuacugucuu.....    9     0    seq
........................................................cuauacaauUuacugucuuu....    1     1    seq
........................................................cuauacaaucuacugucuuu....    323   0    seq
........................................................cuauacaaucuacuguUuuuc...    1     1    seq
........................................................cuauacaaucuacugucuuuc...    297   0    seq
........................................................cuauacaaucuacugucuuucc..    890   0    seq
........................................................cuauacaaucuacugucuuuccu.    21    0    seq
.........................................................uauacaaucuacugucuuu....    5     0    seq
.........................................................uauacaaucuacugucuuuc...    5     0    seq
.........................................................uauacaaucuacugucuuucc..    15    0    seq
.........................................................uauacaaucuacugucuuuccu.    39    0    seq
.........................................................uauacaaucuacugucuuuccua    1     0    seq
..........................................................auacaaucuacugucuuucc..    2     0    seq
..........................................................auacaaucuacugucuuuccu.    6     0    seq
...........................................................uacaaucuacugucuuuccu.    1     0    seq
```
